# Time-to-event analysis mitigates the impact of symptomatic therapy on therapeutic benefit in Parkinson’s disease trials

**DOI:** 10.1038/s41531-025-01041-9

**Published:** 2025-07-01

**Authors:** Gennaro Pagano, Dylan Trundell, Tanya Simuni, Nicola Pavese, Kenneth Marek, Ronald B. Postuma, Nima Shariati, Annabelle Monnet, Emma Moore, Evan W. Davies, Hanno Svoboda, Nathalie Pross, Azad Bonni, Tania Nikolcheva, for the PASADENA Investigators, Tanya Simuni, Claudia Altendorf, Chareyna Anandan, Giulia Andrews, Solène Ansquer, Raphaele Arrouasse, Sana Aslam, Jean-Philippe Azula, Jeanette Baker, Ernest Balaguer Martinez, Shadi Barbu, Kara Bardram, Danny Bega, Helena Bejr-Kasem Marco, Isabelle Benatru, Eve Benchetrit, Felix Bernhard, Amit Besharat, Sagari Bette, Amelie Bichon, Andrew Billnitzer, Sophie Blondeau, Thomas Boraud, Freiderike Borngräber, James Boyd, Kathrin Brockmann, Matthew Brodsky, Ethan Brown, Christof Bruecke, Fabienne Calvas, Monica Canelo, Federico Carbone, Claire Carroll, Laura Casado Fernandez, Catherine Casse-Parrot, Anna Castrioto, Helene Catala, Justine Chan, Samia Cheriet, Anthony Ciabarra, Joseph Classen, Juliana Coleman, Robert Coleman, Yaroslau Compta, Jean-Christophe Corvol, Mariana Cosgaya, Nabila Dahodwala, Philippe Damier, Elodie David, Thomas Davis, Marissa Dean, Berengere Debilly, Janell DeGiorgio, Andres Deik, Laure Delaby, Marie-Helene Delfini, Pascal Derkinderen, Philipp Derost, Maria de Toledo, Lisa Deuel, Ann Marie DiazHernandez, Cameron Dietiker, Karina Dimenshteyn, Julio Dotor, Franck Durif, Jens Ebentheuer, Karla Maria Eggert, Sara Eichau Madueño, Claudia Eickoff, Aaron Ellenbogen, Philipp Ellmerer, Ines Esparragosa Vazquez, Alexandre Eusebio, Siobhan Ewert, John Fang, Danielle Feigenbaum, Frederique Fluchere, Alexandra Foubert-Samier, Marie Fournier, Anne Fradet, Valerie Fraix, Samuel Frank, Franka Fries, Monique Gailitzky, Anne Gaille Corbille, Marisol Gallardó Pérez, Jose Manuel Garcia Morena, Carmen Gasca, Thomas Gasser, Joyce Gibbons, Caroline Giordana, Alicia Gonzalez Martinez, Ira Goodman, Arantza Gorospe, Marie Goubeaud, David Grabli, Mangone Graziella, Stephan Grimaldi, Jeffrey Gross, Raquel Guimaraes-Costa, Andreas Hartmann, Christian Hartmann, Travis Hassell, Srinath Kadimi, Robert Hauser, Antonio Hernandez, Jorge Hernandez-Vara, Günter Höglinger, Christian Homedes, Andrea Horta, Jean-Luc Houeto, Julius Huebl, Jennifer Hui, Stuart Isaacson, Joseph Jankovic, Annette Janzen, Jocelyne Jiao, Maria Jose Marti Domenech, Xavier Joseph, Pat Kaminski, Silja Kannenberg, Jan Kassubek, Maya Katz, Kevin Klos, Shannon Klos, Christopher Kobet, Jennifer Koebert, Patricia Krause, Andrea Kuhn, Jaime Kulisevsky Bojarsky, Rajeev Kumar, Martin Kunz, Lille Kurvits, Kimberly Kwei, Simon Laganiere, Brice Laurens, Johannes Levin, Oren Levy, Peter Le Witt, Gurutz Linazasoro Cristobal, Irene Litvan, Karlo Lizarraga, Katherine Longardner, Rocio Lopez, Lydia Lopez Manzanares, Sara Lucas del Pozo, Maria Rosario Luquin Puido, Nijee Luthra, Kelly Lyons, Sylvia Maass, Gerrit Machetanz, Yolanda Macias, David Maltete, Jorge Uriel Manez Miro, Louis-Laure Mariani, Juan Marin, Kathrin Marini, Ana Marques, Gloria Marti, Saul Martinez, Wassilios Meissner, Sara Meoni, Brit Mollenhauer, Dunia Mon Martinez, Johnson Moon, Elena Moro, Peter Morrison, Christoph Muehlberg, Manpreet Multani, Christine Murphy, Anthony Nicholas, Rajesh Pahwa, Antonio Palasis, Heidi Pape, Neepa Patel, Prity Patel, Marina Peball, Elizabeth Peckham, Terry Peery, Jesus Perez, Rafael Perez Alisa Petit, Elmar Pinkhardt, Werner Poewe, Elsa Pomies, Cecile Preterre, Joseph Quinn, Olivier Rascol, Philippe Remy, Emily Reuther, Irene Richard, Benjamin Roeben, Jost-Julian Rumpf, David Russell, Hayet Salhi, Daniela Samaniego, Alexandra Samier-Foubert, Alvaro Sanchez-Ferro, Emmanuelle Schmitt, Alfons Schnitzler, Oliver Schorr, Julie Schwartzbard, Kerstin Schweyer, Klaus Seppi, Victoria Sergo, Holly Shill, Andrew Siderowf, Umberto Spampinato, Ashok Sriram, Natividad Stover, Caroline Tanner, Arjun Tarakad, Carolyn Taylor, Claire Thalamus, Thomas Toothaker, Nadege Van Blercom, Nora Vanegas-Arrogave, Lydia Vela, Sylvian Vergnet, Tiphaine Vidal, Jonathan Vöglein, Ryan Walsh, Cheryl Waters, Mirko Wegschneider, Endy Weidinger, Caroline Weill, Gregor Wenzel, Tatiana Witjas, Isabel Wurster, Brenton Wright, Milan Zimmermann, Rafael Zuzuarregui, Prasinezumab Study Group, Nima Shariati, Annabelle Monnet, Emma Moore, Hanno Svoboda, Nathalie Pross, Azad Bonni, Tania Nikolcheva, Markus Abt, Atieh Bamdadian, Teresa Barata, Nicholas Barbet, Sara Belli, Frank Boess, Edilio Borroni, Anne Boulay, Markus Britschgi, Valerie Cosson, Christian Czech, Evan Davies, Dennis Deptula, Cheikh Diack, Rachelle Doody, Juergen Dukart, Giulia D’Urso, Sebastian Dziadek, Chris Edgar, Laurent Essioux, Morgan Farell, Rebecca Finch, Paulo Fontoura, Waltraud Gruenbauer, Andrea Hahn, Stefan Holiga, Michael Honer, Shirin Jadidi, Timothy Kilchenmann, Thomas Kremer, Thomas Kustermann, Claire Landsdall, Michael Lindemann, Florian Lipsmeier, Cecile Luzy, Marianne Manchester, Maddalena Marchesi, Ferenc Martenyi, Meret Martin-Facklam, Katerina Mironova, Markus Niggli, Susanne Ostrowitzki, Gennaro Pagano, Benedicte Passemard, Agnes Poirier, Anke Post, Megana Prasad, Benedicte Ricci, Ellen Rose, Daria Rukina, Christoph Sarry, Marzia A. Scelsi, Christine Schubert, Jeff Sevigny, Kaycee Sink, Alexander Strasak, Hannah Staunton, Kirsten I. Taylor, Dylan Trundell, Daniel Umbricht, Lynne Verselis, Annamarie Vogt, Ekaterina Volkova-Volkmar, Cornelia Weber, Silke Weber, Stefano Zanigni

**Affiliations:** 1 Roche Pharma Research and Early Development (pRED), Neuroscience and Rare Diseases Discovery; and Translational Area, Roche Innovation Center Basel, Basel, Switzerland; 2 University of Exeter Medical School, London, UK; 3 Roche Products Ltd, Welwyn Garden City, UK; 4 Department of Neurology, Northwestern University Feinberg School of Medicine, Chicago, IL USA; 5 Clinical Ageing Research Unit, Newcastle University, Newcastle upon Tyne, UK; 6 Institute for Neurodegenerative Disorders, New Haven, CT USA; 7 Department of Neurology, McGill University, and Montreal Neurological Institute, Montreal, Canada; 8 F. Hoffmann-La Roche Ltd, Basel, Switzerland; 9 Roche Diagnostics GmbH, Penzberg, Germany; 10 Berlin Medical University, Neurology Clinic, Campus Charité Mitte, Berlin, Germany; 11 Baylor College of Medicine, Houston, TX USA; 12 Barrow Neurological Institute, Phoenix, AZ USA; 13 Poitiers University Hospital, Poitiers, France; 14 Henri-Mondor University Hospital, Créteil, France; 15 Marseille University Hospital Timone, Marseille, France; 16 University of Vermont, Larner College of Medicine, Burlington, VT USA; 17 General University Hospital of Catalonia, Barcelona, Spain; 18 Quest Research Institute, Farmington Hills, MI USA; 19 Northwestern University, Evanston, IL USA; 20 Policlinica Gipuzkoa Servicio De Neurologia, Gipuzkoa, Spain; 21 Philipps University of Marburg, Neurology Clinic, Marburg, Germany; 22 University of Southern California, Keck Medical Center, Los Angeles, CA USA; 23 Parkinson’s Disease and Movement Disorders Center of Boca Raton, Boca Raton, FL USA; 24 Grenoble Alpes University Michallon Hospital, La Tronche, France; 25 Hospital Pellegrin Bordeaux, Bordeaux, France; 26 Tubingen University Hospital, Tubingen, Germany; 27 Oregon Health & Science University, Portland, OR USA; 28 University of California, San Francisco, CA USA; 29 Toulouse University, Clinical Research Center, Purpan Hospital, Toulouse, France; 30 Goettingen University Medical Center, Paracelsus Elena Klinik Kassel, Goettingen, Germany; 31 Medical University of Innsbruck, Neuroradiology Clinic, Innsbruck, Austria; 32 Compass Research LLC, Orlando, FL USA; 33 De La Princessa University Hospital, Madrid, Spain; 34 Neurology Center of North Orange County, Fullerton, CA USA; 35 Leipzig University, Neurology Clinic and Polyclinic, Leipzig, Germany; 36 Spectrum Health Medical Group, MI, USA; 37 Hospital Clinic Barcelona, Barcelona, Spain; 38 Sorbonne University, Pitié-Salpêtrière University Hospital, Paris, France; 39 University of Pennsylvania, Philadelphia, PA USA; 40 Nantes University, North Laennec University Hospital, Saint-Herblain, France; 41 Nice University, Hospital Pasteur, Nice, France; 42 Vanderbilt University Medical Center, Nashville, TN USA; 43 University of Alabama, UAB Medicine, Birmingham, AL USA; 44 Clermont-Ferrand University Hospital Center, Site Gabriel-Montpied, Clermont-Ferrand, France; 45 Rocky Mountain Movement Disorders Center, Englewood, CO USA; 46 Heinrich Heine University Düsseldorf, University Hospital Düsseldorf, Düsseldorf, Germany; 47 Philips University of Marburg, Marburg, Germany; 48 University of Sevilla, Virgin Macarena University Hospital, Sevilla, Spain; 49 Department of Neurology, University of Navarra, Navarra University Hospital, Navarre, Spain; 50 Beth Israel Deaconess Medical Center, Boston, MA USA; 51 University of Barcelona, The Provincial Clinic Hospital, Barcelona, Spain; 52 University of Madrid, The Alcorcón Foundation University Hospital, Madrid, Spain; 53 Invicro, New Haven, CT USA; 54 Associated Neurologists of Southern Connecticut, P.C., Milford, CT USA; 55 University of South Florida, Parkinson’s Disease and Movement Disorders, Tampa, FL USA; 56 Department of Neurology, Vall d’Hebron Barcelona University Hospital, Barcelona, Spain; 57 Technical University of Munich, The Rechts der Isar Hospital, Munich, Germany; 58 Neurosciences Clinic Institute of the Barcelona Hospital Clinic, Parkinson Disease and Movement Disorders Unit, Barcelona, Spain; 59 Henry Ford Health System, Clarkston, MI USA; 60 University of Ulm, Ulm University Hospital, Clinic for Neurology, Ulm, Germany; 61 The Movement Disorder Clinic of Oklahoma, Tulsa, OK USA; 62 Autonomous University of Barcelona, Hospital de la Santa Creu i Sant Pau, Autoimmune Neurology Unit – Neurology Service, Barcelona, Spain; 63 Columbia University, New York, CU USA; 64 University of California San Diego Altman Clinical and Translational Research Institute, La Jolla, CA USA; 65 University of Rochester Medical Center, Rochester, NY USA; 66 University of Kansas Medical Center, Kansas City, KS USA; 67 University of Rouen Normandy Hospital, Charles Nicolle Parkinson’s Disease Center, Rouen, France; 68 Central Texas Neurology Consultants, Round Rock, TX USA; 69 Aventura Neurologic Associates, Aventura, FL USA; 70 Prothena Corporation plc, Dublin, Ireland

**Keywords:** Parkinson's disease, Parkinson's disease

## Abstract

The use of symptomatic medications represents a challenge for clinical trials of novel medicines designed to slow Parkinson’s disease progression. A time-to-event (TTE) approach using a defined motor progression milestone may mitigate the confounding effect of symptomatic therapy on the Movement Disorders Society-sponsored revision of the Unified Parkinson’s Disease Rating Scale (MDS-UPDRS). This analysis uses prasinezumab- and placebo-treated groups from the PASADENA study to evaluate the impact of symptomatic medications on treatment effects by comparing a TTE approach to a change-from-baseline approach with and without censoring the population upon starting symptomatic therapy. While the TTE approach yielded consistent hazard ratios between censored and non-censored analyses, the estimated difference between treatment arms using the change-from-baseline approach was lower without censoring than with censoring, suggesting a potential masking of prasinezumab treatment effects by symptomatic therapy. Thus, the TTE approach may mitigate the potential confounding effect of symptomatic therapy on MDS-UPDRS Part III.

## Introduction

The use of efficacious standard-of-care symptomatic dopamine replacement therapies represents a major challenge for clinical trials testing novel treatments aimed at slowing Parkinson’s disease (PD) progression in early-stage PD^1–5^. Trials often focus on a treatment-naïve PD population to mitigate the impact of symptomatic medication on clinical rating scales, such as the Movement Disorders Society-sponsored revision of the Unified Parkinson’s Disease Rating Scale (MDS-UPDRS). The MDS-UPDRS is commonly used as an outcome measure; an increase in score indicating worsening as the disease progresses^6,7^. However, as treatment-naïve individuals progress in their PD signs and symptoms and MDS-UPDRS scores increase, they often need to start symptomatic treatments to improve their symptoms, and often this happens within a relatively short period (e.g., 6–12 months). Thus, even though symptomatic medications do not change the course of the disease, any increase/adjustment in medication during the trial is likely to improve the MDS-UPDRS scores, masking the real underlying disease progression. If an experimental drug reduces disease progression and results in less need to change symptomatic medications, an imbalance in medication changes between groups can potentially mask or underestimate the true benefits of the new therapy^8^.

Clinical trials in early-stage PD populations need to have a short duration and/or employ statistical methodologies to mitigate the masking effect of symptomatic treatments; for example, by censoring participants upon starting symptomatic therapies. Having a trial with a short treatment duration limits the interpretation of data and extrapolation of effects to people who are treated with their standard of care over a long period. Employing statistical methodologies, such as censoring participants who start or change symptomatic therapies, is also problematic, given the uncertainty associated with modeling outcomes for visits after the censoring event. Indeed, such approaches may not be accepted by regulators. Thus, solutions that enable clinical trials in early-stage PD populations receiving background standard-of-care medication are required.

In the PASADENA trial, the primary endpoint of change from baseline in MDS-UPDRS Total Score (sum of Parts I, II and III scores) at Week 52 did not reach statistical significance^3^. However, secondary analyses revealed a potential reduction in disease progression with prasinezumab, particularly in subgroups with fast disease progression^4^ and over longer follow-up periods^9^. This suggests that prasinezumab may offer benefits beyond symptomatic relief, but due to the slow progression of PD, these effects may require sensitive analytical approaches to be detected in a short trial. The PASADENA study population thus provides a useful dataset with which to evaluate techniques for mitigating the impact of symptomatic therapies.

Time-to-event (TTE) endpoints have been successfully used in trials for other neurodegenerative diseases, including multiple sclerosis^10^, amyotrophic lateral sclerosis^11,12^ and Alzheimer’s disease^13–15^, and may offer a promising way to measure disease progression in clinical trials for PD. Specifically, if the event is more likely to occur prior to changes in symptomatic therapy regimen (including starting a symptomatic therapy), the treatment effect should be similar regardless of whether or not data are censored on this basis. For a trial in PD, a threshold for meaningful motor progression needs to be established. Horvath et al. (2015) produced estimates of meaningful within-patient improvement and worsening across a range of baseline severities determined by Hoehn and Yahr stage^16^. They followed traditional anchor-based estimation methodology, using the Clinical Global Impression of Improvement (CGI-I) as the anchor measure, producing an estimate of a 5-point worsening (a mean change of 4.63 in patients rated as ‘Minimally worse’ and a cut score of 4.5 using a Receiver Operating Characteristic curve) in an early PD population (Hoehn and Yahr Stage 1‒2)^16^. As these analyses were conducted using MDS-UPDRS Part III data collected in ON medication state, Trundell et al. (2025)^17^ subsequently conducted analyses to estimate the threshold for meaningful within-patient worsening for the MDS-UPDRS Part III score in OFF medication state, using data from the PASADENA trial and the CGI-I as the anchor measure^17^. These analyses also support the use of a 5-point threshold for meaningful within-patient worsening (mean, 4.98 points; median, 5 points)^17^. Furthermore, it was demonstrated that those participants who experienced a motor progression (compared to those who did not) had statistically greater progression on measures of meaningful function, such as MDS-UPDRS Part II^17^. Trundell et al. additionally used a modified Delphi panel to seek clinical consensus on the threshold for clinically meaningful motor progression^17^. Based on the results of the anchor-based analyses in OFF and ON medication, the modified Delphi panel achieved consensus supporting the use of 4‒6 points as a suitable range, and the specific use of 5 points as the progression threshold for MDS-UPDRS Part III in OFF medication state^17^. We hypothesized that using time to a ≥5-point increase on MDS-UPDRS Part III would limit the impact of symptomatic medication on the study outcome because changes in medication are more likely to occur after meaningful motor progression has occurred. Indeed, the change in medication could be in response to the meaningful motor progression.

We tested our hypothesis using data from the PASADENA study^3^, by comparing the change from baseline on MDS-UPDRS Part III using a Mixed-Effects Model for Repeated Measures (MMRM) with a TTE approach (i.e., time to meaningful motor progression). Analyses with and without censoring of data upon starting/changing symptomatic medication were performed to test the hypothesis that treatment effects would be similar independent of the approach to handle change in medication with TTE but not with the MMRM methodology.

## Results

### Participant characteristics

Data from 316 participants comprising the modified intent-to-treat (mITT) population who entered Part 1 of the PASADENA study (the initial double-blind phase or 52-week double-blind treatment period) were included in the TTE analysis. For the MMRM analysis, 309 participants who completed Part 1 and initiated Part 2 (the 52-week blinded dose extension phase where all participants received prasinezumab) were included. The low-dose (1500 mg) and high-dose (4500 mg) prasinezumab groups were pooled for this analysis and baseline demographics and disease characteristics were well-balanced between the placebo- and prasinezumab-treated groups^3^.

### MMRM analysis of MDS-UPDRS Part III

**Fig. 1:**
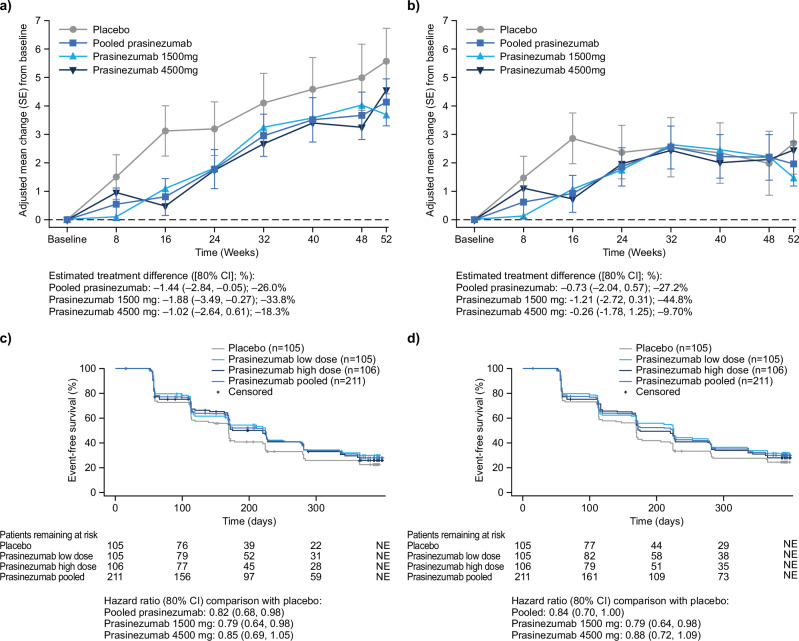
PASADENA Part 1 MDS-UPDRS Part III results. **a** MMRM hypothetical strategy, **b** MMRM treatment policy strategy, **c** TTE ( + 5 points on MDS-UPDRS Part III) hypothetical strategy (Kaplan‒Meier curve), **d** TTE ( + 5 points on MDS-UPDRS Part III) treatment policy strategy (Kaplan‒Meier curve). CI confidence interval, MDS-UPDRS Movement Disorder Society-sponsored revision of the Unified Parkinson’s Disease Rating Scale, MMRM Mixed-effect Model for Repeated Measures, TTE time-to event.

The mean (standard error) change from baseline in MDS-UPDRS Part III score at Week 52 was 5.57 (±0.897) points for the placebo-treated group and 4.12 (±0.646) points for the prasinezumab-treated group when censoring the participants after they started symptomatic therapy (Fig. 1a). The estimated treatment difference was −1.44 points (80% confidence interval [CI]: −2.84, −0.05) (Table 1). Without censoring the participants after they started symptomatic therapy (Fig. 1b), the mean (standard error) change from baseline at Week 52 was 2.68 (±0.841) points for the placebo-treated group and 1.95 (±0.606) points for the prasinezumab-treated group, with an estimated treatment difference of −0.73 points (80% CI: −2.04, 0.57) (Table 1).

**Table 1:** Overview of MDS-UPDRS Part III MMRM and TTE analyses from the PASADENA full Part 1 data snapshot

MDS-UPDRS Part III (“OFF” medication state) at baseline mITT^a^ Placebo (n = 105) vs. prasinezumab pooled^b^ (n = 211)		
Estimand strategy	Hypothetical strategy	Treatment policy strategy
Least square mean ± SE from MMRM at Week 52 [80% CI]	Placebo (n = 76): 5.57 ± 0.897 [4.42, 6.72] Prasinezumab pooled (n = 147): 4.12 ± 0.646 [3.29, 4.95]	Placebo (n = 105): 2.68 ± 0.841 [1.60, 3.76] Prasinezumab pooled (n = 205): 1.95 ± 0.606 [1.17, 2.73]
Difference in least square mean [80% CI] at Week 52	−1.44 [ − 2.84, -0.05]	−0.73 [−2.04, 0.57]
Hazard ratio from TTE [80% CI] (using Cox proportional hazard model)	0.82 [0.68, 0.98] (N = 316; 2:1 prasinezumab to placebo ratio)	0.84 [0.70, 1.00] (N = 316; 2:1 prasinezumab to placebo ratio)

^a^mITT population enrolled in Part 1 of PASADENA.

^b^Pooled prasinezumab doses, either 1500 mg or 4500 mg, for 52 weeks.

*CI* confidence interval, *MDS-UPDRS* Movement Disorder Society-sponsored revision of the Unified Parkinson’s Disease Rating Scale, *mITT* modified intent-to-treat, *MMRM* Mixed-effect Model for Repeated Measures, *SE* standard error, *TTE* time-to-event.

### TTE analysis

The hazard ratio for time to a 5-point increase in MDS-UPDRS Part III score, comparing the placebo-treated group to the prasinezumab-treated group, was 0.82 (80% CI: 0.68, 0.98) when censoring the participants after they started symptomatic therapy (Fig. 1c and Table 1) and 0.84 (80% CI: 0.70, 1.00) without censoring (Fig. 1d and Table 1).

These analyses were also consistent in the comparison of the early-start and delayed-start groups at Week 104 (Supplementary Table S1 and Supplementary Figure S1).

### Impact of symptomatic therapy initiation

During the first 52 weeks, the proportion of events where a 5-point or greater worsening in MDS-UPDRS Part III score was observed before starting symptomatic PD therapy was 97% in the placebo-treated group and 96% in the prasinezumab-treated group.

## Discussion

The presented analyses highlight the complexities of evaluating potential disease-modifying therapies in early-stage PD. Our findings provide evidence that traditional continuous change from baseline analyses using uncensored data might underestimate disease progression and potentially mask the effects of novel treatments aimed at slowing disease progression. Whilst the modeled change following censoring using an MMRM approach could have overestimated the treatment effect, the lack of consistency observed with and without censoring is concerning and supportive of our hypothesis. Furthermore, our results demonstrated greater consistency in hazard ratios between the censored and non-censored analyses and narrower CIs for the TTE approach, compared to the MMRM approach. Indeed, most participants in the PASADENA trial experienced a meaningful motor progression milestone (≥5-point worsening in MDS-UPDRS Part III) before starting their symptomatic therapy. This suggests that TTE endpoints may offer a more reliable approach for assessing disease-modifying therapies in the context of clinical trials on top of standard of care, where symptomatic treatment adjustments may occur.

The start of symptomatic therapy reduced the measurable disease progression (increase in MDS-UPDRS Part III points at Week 52) by 2.89 points in the placebo group (5.57 points in the censoring analysis minus 2.68 points in the analysis without censoring), which is equivalent to 52% less measurable progression over the treatment period. The observed attenuation of treatment effects in the MMRM analysis further supports the hypothesis that symptomatic therapy initiation or changes can mask the potential benefits of disease-modifying therapies such as prasinezumab. This phenomenon poses significant challenges for clinical trial design in PD. As the disease progresses, patients will likely require adjustments in their symptomatic medication regimens, potentially confounding the assessment of disease-modifying effects and requiring larger and longer studies. However, while increasing the dosage or frequency of symptomatic medications can temporarily alleviate motor symptoms, this approach has inherent limitations. Dopamine replacement therapies do not alter the underlying disease course, and over-reliance on escalating doses can lead to complications, such as motor fluctuations, dyskinesias, and other side effects, ultimately diminishing the patient’s quality of life^18^. Therefore, assessing the true effect of a disease-modifying therapy early on is critical, even in the presence of symptomatic medications. By demonstrating the potential to delay or reduce the need for escalating symptomatic treatments, a disease-modifying therapy offers an important advantage beyond simply managing current symptoms. This translates into a potential preservation of therapeutic options for later stages, a critical consideration for patients facing a progressive disease like PD.

Focusing clinical trials exclusively on advanced PD populations, where symptomatic medication changes are no longer feasible, also poses significant challenges. In these later stages, motor fluctuations, non-motor symptoms, and comorbidities are often more prevalent and severe, introducing considerable variability and confounding factors into study outcomes. The complex interplay of these factors can make it difficult to isolate and accurately measure the specific impact of a disease-modifying therapy. Furthermore, patients with advanced PD may have limited remaining capacity to further slow their neurodegeneration and motor progression, potentially underestimating the true benefit of a disease-modifying intervention. Therefore, studying early-stage PD populations, where the potential for disease modification is greater, provides a clearer picture of the therapy’s impact on slowing disease progression.

Another core advantage of a TTE endpoint is that it is easier to interpret than a mean change from baseline comparison, given that its meaningfulness to patients is directly linked to the meaningfulness of the event, with inherent patient-centricity and interpretability. Unlike mean changes on continuous rating scales, which can be abstract and difficult for patients to grasp, TTE outcomes focus on discrete, clinically relevant events that directly impact a patient’s life. In the context of PD, the event of reaching a 5-point worsening in MDS-UPDRS Part III signifies a tangible decline in motor function^17^, often translating to noticeable difficulties in daily activities and a reduced quality of life.

This shift in focus from numerical scores to meaningful events aligns with the growing emphasis on patient-centered care. By prioritizing outcomes that resonate with patients’ lived experiences, clinical trials can better capture the true impact of therapeutic interventions and empower patients to make informed decisions about their treatment options. Furthermore, the interpretability of TTE outcomes facilitates communication between healthcare providers and patients. Discussing the likelihood of reaching a specific milestone, such as a 5-point decline in motor function, is more intuitive and relatable than explaining changes in average scores. This clarity can enhance shared decision-making, as patients and clinicians can engage in meaningful conversations about the risks and benefits of different treatment options based on concrete, patient-relevant outcomes.

Future research could explore other patient-centric TTE endpoints in addition to the 5-point worsening threshold. These could include events related to non-motor symptoms, such as cognitive decline or sleep disturbances, or milestones related to functional independence and quality of life. This milestone-based strategy to monitor PD progression has been implemented in the Parkinson’s Progression Markers Initiative study^19^. By incorporating a broader range of patient-relevant outcomes, clinical trials can provide a more comprehensive understanding of the impact of disease-modifying therapies on the overall well-being of individuals with PD.

The generalizability of our findings may be limited by the specific design of the PASADENA trial. Since the participants were discouraged from starting symptomatic therapy during the 52-week, Part 1, double-blind, placebo-controlled period unless deemed clinically essential, most participants experienced motor worsening before starting symptomatic therapy, which may not be replicable in future clinical trials with different study designs or population characteristics. Additionally, the lack of imbalance in the PASADENA trial in the proportion of events reached prior to starting symptomatic therapy between treatment groups (97% in placebo-treated group and 96% in the prasinezumab-treated group) could potentially influence the relative robustness of the TTE and MMRM approaches in future trials. Thus, while the PASADENA trial provides valuable insights into the potential of TTE analyses, its specific design — where most participants experienced motor worsening before initiating symptomatic therapy — may not fully reflect the complexities of real-world clinical practice. In routine care, patients with early-stage PD often start or adjust symptomatic medications as part of their standard management, and these adjustments may occur at different time points and with varying frequencies.

These findings raise the question of how a TTE analysis would perform in a broader population with more heterogeneous medication patterns. Thus, it will be crucial to assess whether the observed advantages of TTE over MMRM in mitigating the impact of symptomatic therapy changes hold true when treatment adjustments occur organically and less predictably throughout the study period. To bridge this gap, future research should prioritize studies designed to address the generalizability of TTE analyses in real-world settings. For example, by conducting trials in larger, more diverse cohorts of early-stage PD patients with varying baseline characteristics, disease severity, and medication regimens. This would allow for a more comprehensive evaluation of the TTE approach across different patient subgroups and treatment scenarios. Complementing randomized controlled trials with observational studies that track real-world medication use and disease progression in early PD would provide valuable insights into the natural history of the disease and the impact of symptomatic therapy adjustments on motor outcomes. Employing modeling and simulation techniques to explore the robustness of TTE analysis under different assumptions about treatment patterns, adherence, and disease progression rates could help identify potential biases and limitations of the approach and inform future trial design.

Another potential limitation of a TTE approach is the dichotomization of the MDS-UPDRS, a continuous scale, which can lead to a potential disadvantage of reduced accuracy and pose the question of the variability around the threshold and its acceptability. The selection of a 5-point worsening in MDS-UPDRS Part III as the threshold for meaningful motor progression in our TTE analysis was a critical decision that balances clinical relevance with statistical considerations. This threshold is supported by a growing body of evidence suggesting that a change of 5 points or more on the MDS-UPDRS Part III represents a clinically meaningful decline in motor function, both in the “ON” and “OFF” medication states^16,17^. This threshold aligns with expert consensus and has been shown to correlate with patient-reported quality-of-life measures. Moreover, the 5-point threshold offers a practical advantage in clinical trials, since it represents a significant enough change to be detectable within a reasonable timeframe, allowing for efficient study design and data analysis. As indicated above, although the dichotomization of a continuous scale like MDS-UPDRS Part III simplifies the analysis and interpretation of results, it inevitably leads to some loss of information. By categorizing patients into two groups (those who have reached the threshold and those who have not), we may overlook the nuanced variations in disease progression that occur below the threshold. This could potentially affect the sensitivity and specificity of the TTE analysis. For instance, patients who experience a 4-point worsening may still be experiencing clinically meaningful decline, but they would be categorized as not having reached the event in the TTE analysis. To address this limitation, future research could explore alternative approaches to threshold selection and analysis. For example, by evaluating the impact of different thresholds on the TTE analysis and comparing the results to assess the robustness of the findings, or by utilizing time-varying thresholds that adjust based on individual patient characteristics or disease progression patterns. Indeed, in PASADENA, using 4 points as the threshold instead of 5 points produced comparable results (i.e., a similar magnitude of effect between the estimand strategies); however, with 6 points the treatment effect was larger under the hypothetical strategy than the treatment policy strategy, due to an increase in the proportion of patients changing their symptomatic therapy regimen prior to the event (data not shown).

The choice of a threshold and the decision to dichotomize a continuous scale involve a trade-off between clinical relevance, statistical considerations, and practical feasibility. The 5-point threshold represents a pragmatic and clinically meaningful choice, but future research should continue to refine our understanding of how best to measure and analyze disease progression in PD using TTE endpoints and explore the statistical properties of TTE analysis in more depth, comparing different modeling approaches and censoring mechanisms. Additionally, developing methods to account for potential heterogeneity in treatment effects across patient subgroups and exploring the impact of different baseline characteristics on TTE outcomes could further enhance the understanding of disease progression in PD. By rigorously evaluating the statistical considerations and robustness of TTE analysis, researchers can strengthen the evidence base for this approach and ensure its appropriate application in clinical trials for PD and other neurodegenerative diseases.

The PASADENA study and its subsequent TTE analysis have opened up a promising avenue for evaluating disease-modifying therapies in PD. However, while the findings of this study are encouraging, further exploration and validation are necessary to solidify the role of TTE endpoints in clinical trials. The ongoing PADOVA study (NCT04777331), designed to specifically test the efficacy of prasinezumab using the TTE approach described here, represents a critical step in this direction. By employing a similar methodology in an early PD population on standard of care, PADOVA has the potential to confirm the validity and generalizability of TTE analysis as a primary endpoint. Positive results from this trial could revolutionize the design of future clinical trials in PD, paving the way for a more patient-centric and clinically relevant evaluation of disease-modifying therapies.

In conclusion, despite acknowledged limitations, our results suggest a potential advantage of TTE over MMRM in mitigating the impact of symptomatic therapy changes on motor outcomes in early-stage PD trials. Further research is needed to validate these findings in larger and more representative populations (e.g., evaluating real-life early PD populations using different symptomatic therapy patterns) and to explore alternative strategies for minimizing the confounding effects of symptomatic treatment adjustments. Even if the journey towards establishing TTE analysis as a standard tool in PD clinical trials is still ongoing, the potential rewards are significant. By embracing this innovative approach, we can accelerate the development of effective treatments, empower patients with meaningful information about their disease, and ultimately improve the lives of those affected by PD.

## Methods

### Participants

Data were obtained from the mITT population of the PASADENA study (NCT03100149), an ongoing multicenter, Phase 2, double-blind, placebo-controlled trial evaluating the safety and efficacy of prasinezumab in early-stage PD. Eligible participants had a disease duration of ≤2 years, Hoehn and Yahr Stage I or II, and were either drug-naïve or on stable monoamine oxidase type B inhibitor monotherapy. Of the 316 participants included in this study, the mean (standard deviation) age was 59.9 (9.1) years, 213 (67.4%) were male, and 78 (24.7%) were Hoehn and Yahr Stage I.

The trial and recruitment materials were approved by institutional review boards or ethics committees at each trial site. The trial was conducted according to the principles of the Declaration of Helsinki and Good Clinical Practice guidelines. All the participants provided written informed consent before undergoing any trial-specific screening tests or evaluations.

### Study design

The study design consisted of three parts:

1. Part 1: 52-week double-blind treatment period (prasinezumab or placebo).
2. Part 2: 52-week blinded extension (all participants received prasinezumab) followed by a 12-week treatment-free period.
3. Part 3 (ongoing): Open-label extension with all participants receiving prasinezumab.

During the initial double-blind phase (Part 1), participants were expected to refrain from initiating symptomatic PD therapy.

### Quantification and statistical analysis: statistical models

*Post-hoc* analyses were conducted on the Part 1 double-blind phase of the trial, to compare the prasinezumab-treated group (prasinezumab for 52 weeks) and placebo-treated group (placebo for 52 weeks) using two models:

1. MMRM: To estimate the change from baseline in MDS-UPDRS Part III score at Week 52.
2. Cox proportional hazards model: To estimate the hazard ratio for the time to a 5-point increase in MDS-UPDRS Part III score.

A MMRM was used for the longitudinal endpoints including covariates: treatment arm, background therapy at baseline, age, sex, dopamine transporter-single-photon emission computed tomography (DaT-SPECT), contralateral putamen binding ratio at baseline, the visit (as a categorical factor), a group-by-visit interaction and the baseline endpoint. Within each participant, the model incorporates an unstructured variance–covariance matrix for the random error terms. Adjusted mean differences were extracted from the MMRM. Disease progression curves in each treatment arm were estimated using Kaplan–Meier methodology. The treatment effect was quantified via a hazard ratio, computed from a stratified Cox proportional-hazards regression model, including a 95% CI. The Cox model was adjusted on the randomization stratification factors with background therapy at baseline, age, sex, DaT-SPECT contralateral putamen binding ratio at baseline.

### Estimands and robustness assessment

Two estimands were used to assess the robustness of the results:

- Hypothetical strategy: Included data from all randomized participants until initiation or change in symptomatic therapy
- Treatment policy strategy: Included all data regardless of changes in symptomatic therapy.

### Additional analyses

The proportion of participants experiencing a 5-point or greater worsening in MDS-UPDRS Part III score prior to the initiation or change of PD symptomatic therapy was assessed for both the prasinezumab-treated and placebo-treated groups during the first year of the trial (Part 1). All *post-hoc* analyses were also performed on the Part 2 blinded extension phase of the trial, to compare the early-start group (prasinezumab for 104 weeks) and delayed-start group (placebo for 52 weeks, and then prasinezumab for 52 weeks).

## Supplementary information

Supplement

## Data Availability

Qualified researchers may request access to individual patient-level data through the clinical study data request platform (https://vivli.org/). Further details on Roche’s criteria for eligible studies are available at https://vivli.org/members/ourmembers/. For further details on Roche’s Global Policy on the Sharing of Clinical Information and how to request access to related clinical study documents, see: https://www.roche.com/research_and_development/who_we_are_how_we_work/clinical_trials/our_commitment_to_data_sharing.htm.
