# Author Correction: Time-to-event analysis mitigates the impact of symptomatic therapy on therapeutic benefit in Parkinson’s disease trials

**DOI:** 10.1038/s41531-025-01103-y

**Published:** 2025-08-13

**Authors:** Gennaro Pagano, Dylan Trundell, Tanya Simuni, Nicola Pavese, Kenneth Marek, Ronald B. Postuma, Nima Shariati, Annabelle Monnet, Emma Moore, Evan W. Davies, Hanno Svoboda, Nathalie Pross, Azad Bonni, Tania Nikolcheva, for the PASADENA Investigators, Tanya Simuni, Claudia Altendorf, Chareyna Anandan, Giulia Andrews, Solène Ansquer, Raphaele Arrouasse, Sana Aslam, Jean-Philippe Azula, Jeanette Baker, Ernest Balaguer Martinez, Shadi Barbu, Kara Bardram, Danny Bega, Helena Bejr-Kasem Marco, Isabelle Benatru, Eve Benchetrit, Felix Bernhard, Amit Besharat, Sagari Bette, Amelie Bichon, Andrew Billnitzer, Sophie Blondeau, Thomas Boraud, Freiderike Borngräber, James Boyd, Kathrin Brockmann, Matthew Brodsky, Ethan Brown, Christof Bruecke, Fabienne Calvas, Monica Canelo, Federico Carbone, Claire Carroll, Laura Casado Fernandez, Catherine Casse-Parrot, Anna Castrioto, Helene Catala, Justine Chan, Samia Cheriet, Anthony Ciabarra, Joseph Classen, Juliana Coleman, Robert Coleman, Yaroslau Compta, Jean-Christophe Corvol, Mariana Cosgaya, Nabila Dahodwala, Philippe Damier, Elodie David, Thomas Davis, Marissa Dean, Berengere Debilly, Janell DeGiorgio, Andres Deik, Laure Delaby, Marie-Helene Delfini, Pascal Derkinderen, Philipp Derost, Maria de Toledo, Lisa Deuel, Ann Marie DiazHernandez, Cameron Dietiker, Karina Dimenshteyn, Julio Dotor, Franck Durif, Jens Ebentheuer, Karla Maria Eggert, Sara Eichau Madueño, Claudia Eickoff, Aaron Ellenbogen, Philipp Ellmerer, Ines Esparragosa Vazquez, Alexandre Eusebio, Siobhan Ewert, John Fang, Danielle Feigenbaum, Frederique Fluchere, Alexandra Foubert-Samier, Marie Fournier, Anne Fradet, Valerie Fraix, Samuel Frank, Franka Fries, Monique Gailitzky, Anne Gaille Corbille, Marisol Gallardó Pérez, Jose Manuel Garcia Morena, Carmen Gasca, Thomas Gasser, Joyce Gibbons, Caroline Giordana, Alicia Gonzalez Martinez, Ira Goodman, Arantza Gorospe, Marie Goubeaud, David Grabli, Mangone Graziella, Stephan Grimaldi, Jeffrey Gross, Raquel Guimaraes-Costa, Andreas Hartmann, Christian Hartmann, Travis Hassell, Srinath Kadimi, Robert Hauser, Antonio Hernandez, Jorge Hernandez-Vara, Günter Höglinger, Christian Homedes, Andrea Horta, Jean-Luc Houeto, Julius Huebl, Jennifer Hui, Stuart Isaacson, Joseph Jankovic, Annette Janzen, Jocelyne Jiao, Maria Jose Marti Domenech, Xavier Joseph, Pat Kaminski, Silja Kannenberg, Jan Kassubek, Maya Katz, Kevin Klos, Shannon Klos, Christopher Kobet, Jennifer Koebert, Patricia Krause, Andrea Kuhn, Jaime Kulisevsky Bojarsky, Rajeev Kumar, Martin Kunz, Lille Kurvits, Kimberly Kwei, Simon Laganiere, Brice Laurens, Johannes Levin, Oren Levy, Peter Le Witt, Gurutz Linazasoro Cristobal, Irene Litvan, Karlo Lizarraga, Katherine Longardner, Rocio Lopez, Lydia Lopez Manzanares, Sara Lucas del Pozo, Maria Rosario Luquin Puido, Nijee Luthra, Kelly Lyons, Sylvia Maass, Gerrit Machetanz, Yolanda Macias, David Maltete, Jorge Uriel Manez Miro, Louis-Laure Mariani, Juan Marin, Kathrin Marini, Ana Marques, Gloria Marti, Saul Martinez, Wassilios Meissner, Sara Meoni, Brit Mollenhauer, Dunia Mon Martinez, Johnson Moon, Elena Moro, Peter Morrison, Christoph Muehlberg, Manpreet Multani, Christine Murphy, Anthony Nicholas, Rajesh Pahwa, Antonio Palasis, Heidi Pape, Neepa Patel, Prity Patel, Marina Peball, Elizabeth Peckham, Terry Peery, Jesus Perez, Rafael Perez Alisa Petit, Elmar Pinkhardt, Werner Poewe, Elsa Pomies, Cecile Preterre, Joseph Quinn, Olivier Rascol, Philippe Remy, Emily Reuther, Irene Richard, Benjamin Roeben, Jost-Julian Rumpf, David Russell, Hayet Salhi, Daniela Samaniego, Alexandra Samier-Foubert, Alvaro Sanchez-Ferro, Emmanuelle Schmitt, Alfons Schnitzler, Oliver Schorr, Julie Schwartzbard, Kerstin Schweyer, Klaus Seppi, Victoria Sergo, Holly Shill, Andrew Siderowf, Umberto Spampinato, Ashok Sriram, Natividad Stover, Caroline Tanner, Arjun Tarakad, Carolyn Taylor, Claire Thalamus, Thomas Toothaker, Nadege Van Blercom, Nora Vanegas-Arrogave, Lydia Vela, Sylvian Vergnet, Tiphaine Vidal, Jonathan Vöglein, Ryan Walsh, Cheryl Waters, Mirko Wegschneider, Endy Weidinger, Caroline Weill, Gregor Wenzel, Tatiana Witjas, Isabel Wurster, Brenton Wright, Milan Zimmermann, Rafael Zuzuarregui, Prasinezumab Study Group, Nima Shariati, Annabelle Monnet, Emma Moore, Hanno Svoboda, Nathalie Pross, Azad Bonni, Tania Nikolcheva, Markus Abt, Atieh Bamdadian, Teresa Barata, Nicholas Barbet, Sara Belli, Frank Boess, Edilio Borroni, Anne Boulay, Markus Britschgi, Valerie Cosson, Christian Czech, Evan Davies, Dennis Deptula, Cheikh Diack, Rachelle Doody, Juergen Dukart, Giulia D’Urso, Sebastian Dziadek, Chris Edgar, Laurent Essioux, Morgan Farell, Rebecca Finch, Paulo Fontoura, Waltraud Gruenbauer, Andrea Hahn, Stefan Holiga, Michael Honer, Shirin Jadidi, Timothy Kilchenmann, Thomas Kremer, Thomas Kustermann, Claire Landsdall, Michael Lindemann, Florian Lipsmeier, Cecile Luzy, Marianne Manchester, Maddalena Marchesi, Ferenc Martenyi, Meret Martin-Facklam, Katerina Mironova, Markus Niggli, Susanne Ostrowitzki, Gennaro Pagano, Benedicte Passemard, Agnes Poirier, Anke Post, Megana Prasad, Benedicte Ricci, Ellen Rose, Daria Rukina, Christoph Sarry, Marzia A. Scelsi, Christine Schubert, Jeff Sevigny, Kaycee Sink, Alexander Strasak, Hannah Staunton, Kirsten I. Taylor, Dylan Trundell, Daniel Umbricht, Lynne Verselis, Annamarie Vogt, Ekaterina Volkova-Volkmar, Cornelia Weber, Silke Weber, Stefano Zanigni

**Affiliations:** 1 Roche Pharma Research and Early Development (pRED), Neuroscience and Rare Diseases Discovery; and Translational Area, Roche Innovation Center Basel, Basel, Switzerland; 2 University of Exeter Medical School, London, UK; 3 Roche Products Ltd, Welwyn Garden City, UK; 4 Department of Neurology, Northwestern University Feinberg School of Medicine, Chicago, IL USA; 5 Clinical Ageing Research Unit, Newcastle University, Newcastle upon Tyne, UK; 6 Institute for Neurodegenerative Disorders, New Haven, CT USA; 7 Department of Neurology, McGill University, and Montreal Neurological Institute, Montreal, Canada; 8 F. Hoffmann-La Roche Ltd, Basel, Switzerland; 9 Roche Diagnostics GmbH, Penzberg, Germany; 10 Berlin Medical University, Neurology Clinic, Campus Charité Mitte, Berlin, Germany; 11 Baylor College of Medicine, Houston, TX USA; 12 Barrow Neurological Institute, Phoenix, AZ USA; 13 Poitiers University Hospital, Poitiers, France; 14 Henri-Mondor University Hospital, Créteil, France; 15 Marseille University Hospital Timone, Marseille, France; 16 University of Vermont, Larner College of Medicine, Burlington, VT USA; 17 General University Hospital of Catalonia, Barcelona, Spain; 18 Quest Research Institute, Farmington Hills, MI USA; 19 Northwestern University, Evanston, IL USA; 20 Policlinica Gipuzkoa Servicio De Neurologia, Gipuzkoa, Spain; 21 Philipps University of Marburg, Neurology Clinic, Marburg, Germany; 22 University of Southern California, Keck Medical Center, Los Angeles, CA USA; 23 Parkinson’s Disease and Movement Disorders Center of Boca Raton, Boca Raton, FL USA; 24 Grenoble Alpes University Michallon Hospital, La Tronche, France; 25 Hospital Pellegrin Bordeaux, Bordeaux, France; 26 Tubingen University Hospital, Tubingen, Germany; 27 Oregon Health & Science University, Portland, OR USA; 28 University of California, San Francisco, CA USA; 29 Toulouse University, Clinical Research Center, Purpan Hospital, Toulouse, France; 30 Goettingen University Medical Center, Paracelsus Elena Klinik Kassel, Goettingen, Germany; 31 Medical University of Innsbruck, Neuroradiology Clinic, Innsbruck, Austria; 32 Compass Research LLC, Orlando, FL USA; 33 De La Princessa University Hospital, Madrid, Spain; 34 Neurology Center of North Orange County, Fullerton, CA USA; 35 Leipzig University, Neurology Clinic and Polyclinic, Leipzig, Germany; 36 Spectrum Health Medical Group, MI, USA; 37 Hospital Clinic Barcelona, Barcelona, Spain; 38 Sorbonne University, Pitié-Salpêtrière University Hospital, Paris, France; 39 University of Pennsylvania, Philadelphia, PA USA; 40 Nantes University, North Laennec University Hospital, Saint-Herblain, France; 41 Nice University, Hospital Pasteur, Nice, France; 42 Vanderbilt University Medical Center, Nashville, TN USA; 43 University of Alabama, UAB Medicine, Birmingham, AL USA; 44 Clermont-Ferrand University Hospital Center, Site Gabriel-Montpied, Clermont-Ferrand, France; 45 Rocky Mountain Movement Disorders Center, Englewood, CO USA; 46 Heinrich Heine University Düsseldorf, University Hospital Düsseldorf, Düsseldorf, Germany; 47 Philips University of Marburg, Marburg, Germany; 48 University of Sevilla, Virgin Macarena University Hospital, Sevilla, Spain; 49 Department of Neurology, University of Navarra, Navarra University Hospital, Navarre, Spain; 50 Beth Israel Deaconess Medical Center, Boston, MA USA; 51 University of Barcelona, The Provincial Clinic Hospital, Barcelona, Spain; 52 University of Madrid, The Alcorcón Foundation University Hospital, Madrid, Spain; 53 Invicro, New Haven, CT USA; 54 Associated Neurologists of Southern Connecticut, P.C., Milford, CT USA; 55 University of South Florida, Parkinson’s Disease and Movement Disorders, Tampa, FL USA; 56 Department of Neurology, Vall d’Hebron Barcelona University Hospital, Barcelona, Spain; 57 Technical University of Munich, The Rechts der Isar Hospital, Munich, Germany; 58 Neurosciences Clinic Institute of the Barcelona Hospital Clinic, Parkinson Disease and Movement Disorders Unit, Barcelona, Spain; 59 Henry Ford Health System, Clarkston, MI USA; 60 University of Ulm, Ulm University Hospital, Clinic for Neurology, Ulm, Germany; 61 The Movement Disorder Clinic of Oklahoma, Tulsa, OK USA; 62 Autonomous University of Barcelona, Hospital de la Santa Creu i Sant Pau, Autoimmune Neurology Unit – Neurology Service, Barcelona, Spain; 63 Columbia University, New York, CU USA; 64 University of California San Diego Altman Clinical and Translational Research Institute, La Jolla, CA USA; 65 University of Rochester Medical Center, Rochester, NY USA; 66 University of Kansas Medical Center, Kansas City, KS USA; 67 University of Rouen Normandy Hospital, Charles Nicolle Parkinson’s Disease Center, Rouen, France; 68 Central Texas Neurology Consultants, Round Rock, TX USA; 69 Aventura Neurologic Associates, Aventura, FL USA; 70 Prothena Corporation plc, Dublin, Ireland

**Keywords:** Parkinson's disease, Parkinson's disease

*Correction to*: npj Parkinson’s Disease (2025) **11**:193; 10.1038/s41531-025-01041-9; published online 01 July 2025

In this article the PASADENA Investigators member Jan Kassubek was incorrectly written as R. Jan Kassubek. The original article has been corrected.

