# Supplementary material for: Time-to-event analysis mitigates the impact of symptomatic therapy on therapeutic benefit in Parkinson’s disease trials
Source: NPJ Parkinsons Dis. 2025 Jul 1;11:193. doi: 10.1038/s41531-025-01041-9 (PMC12214479; doi:10.1038/s41531-025-01041-9)

Supplemental Information

Supplementary Table S1. Overview of MDS-UPDRS Part III MMRM and TTE analyses from the PASADENA Part 2 data snapshot

MDS UPDRS Part III ("OFF" medication state) at baseline mITT[a]<br>Delayed start[b] (n=105) versus early start[c] (n=204)		
Estimand strategy	Hypothetical strategy	Treatment policy strategy
Least square mean ± SE from MMRM at Week 104 [80% CI]	Delayed start (n=27):<br>10.59 ± 1.41 [8.77, 12.40]<br><br>Early start (n=55):<br>8.42 ± 1.00 [7.13, 9.70]	Delayed start (n=92):<br>3.55 ± 0.96 [2.31, 4.79]<br><br>Early start (n=170):<br>2.72 ± 0.71 [1.80, 3.63]
Difference in least square mean [80% CI] at Week 104	−2.17 [−4.37, 0.03]	−0.83 [−2.33, 0.67]
Hazard ratio from TTE [80% CI] (using Cox proportional hazard model)	0.82 [0.69, 0.98]<br>(N=316; 2:1 prasinezumab to placebo ratio)	0.77 [0.65, 0.91]<br>(N=316; 2:1 prasinezumab to placebo ratio)

[a]mITT population enrolled in Part 2 of PASADENA;

[b]Delayed-start group; received placebo for the first 52 weeks (PASADENA Part 1) and then prasinezumab 1500 mg or 4500 mg between Weeks 56 and 104;

[c]Early-start group; received prasinezumab 1500 mg or 4500 mg for 104 weeks

CI, confidence interval; MDS-UPDRS, Movement Disorder Society-sponsored revision of the Unified Parkinson's Disease Rating Scale; mITT, modified intent-to-treat; MMRM, Mixed-effect Model for Repeated Measures; SE, standard error; TTE, time to event.

Supplementary Figure S1. PASADENA Part 2 MDS-UPDRS Part III results for: (A) MMRM hypothetical strategy, (B) MMRM treatment policy strategy, (C) TTE (+5 points on MDS-UPDRS Part III) hypothetical strategy (Kaplan–Meier curve), and (D) TTE (+5 points on MDS-UPDRS Part III) treatment policy strategy (Kaplan–Meier curve). CI, confidence interval; MDS-UPDRS, Movement Disorder Society-sponsored revision of the Unified Parkinson's Disease Rating Scale; MMRM, Mixed-effect Model for Repeated Measures; SE, standard error; TTE, time to event.

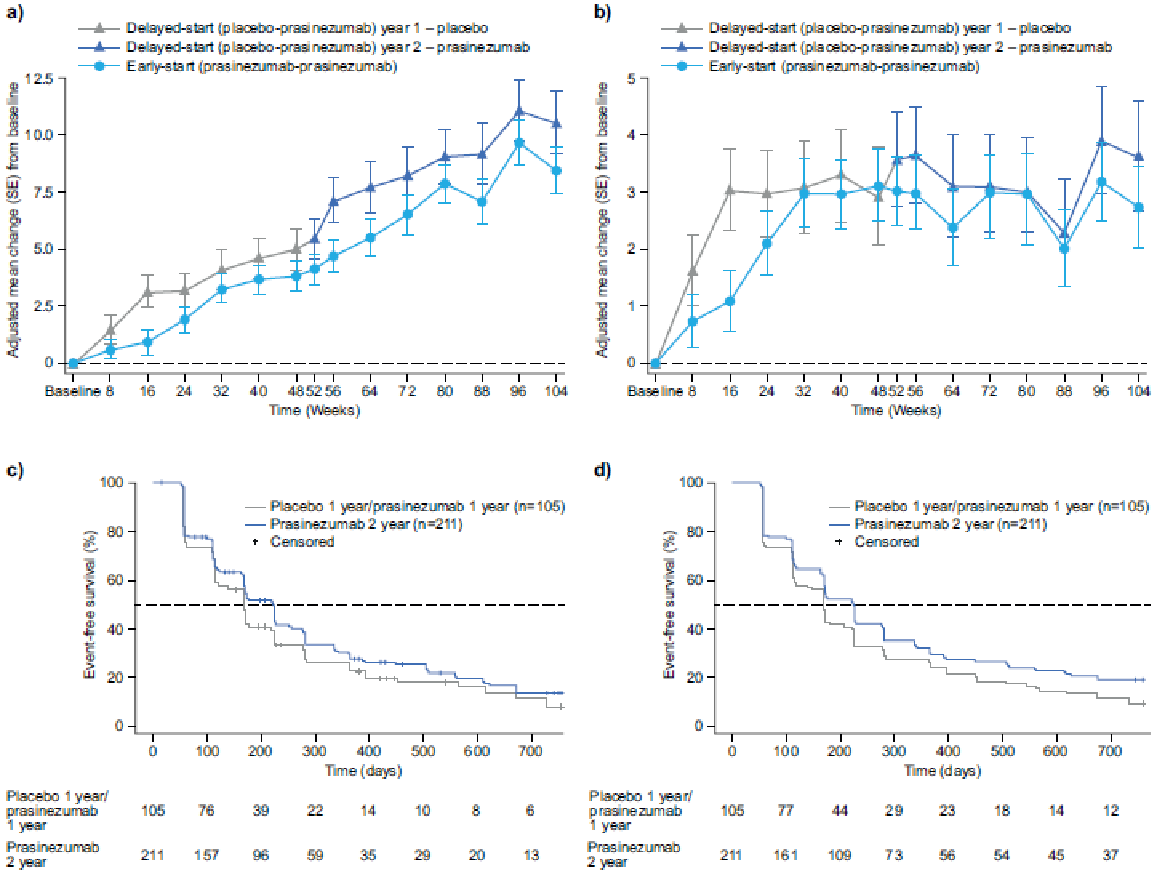

Supplement: Supplementary file 1 — Supplement [file 41531_2025_1041_MOESM1_ESM.pdf]
